# Nurses’ Adoption, Perceived Usability, and Satisfaction with an Updated Electronic Handover Page Within the Electronic Medical Record: A Mixed-Methods Study

**DOI:** 10.3390/nursrep15100369

**Published:** 2025-10-15

**Authors:** Rebecca Miriam Jedwab, Anthony T. Pham, Yixin Qu, Rebecca Brook, Joanne Foster, James-Norbert Garduce, Siwen Li, Jane M. Smith, Naomi Dobroff

**Affiliations:** 1 Nursing and Midwifery Informatics, Department of Clinical Informatics, Digital and Information Division, Monash Health, Clayton, Melbourne, VIC 3168, Australia; anthony.pham@monashhealth.org (A.T.P.); yixin.qu@monashhealth.org (Y.Q.); rebecca.brook@monashhealth.org (R.B.); joanne.foster@monashhealth.org (J.F.); james-norbert.garduce@monashhealth.org (J.-N.G.); siwen.li@monashhealth.org (S.L.); jane.smith3@monashhealth.org (J.M.S.); naomi.dobroff@monashhealth.org (N.D.); 2 School of Nursing and Midwifery, Deakin University, Burwood, Melbourne, VIC 3125, Australia

**Keywords:** attitude to computers, electronic health records, patient handoff, nursing staff, hospital, user–computer interface

## Abstract

**Background/Objective:** Clinical handover of patient information is a key component of patient care in hospitals. Nurses use a structured framework to minimise communication errors. Electronic Medical Record (EMR) systems can support patient safety and clinical handover with contemporaneous documentation. The aim of this study was to evaluate nurses’ adoption, perceived usability, and satisfaction with an updated handover page within the EMR. **Methods:** A pre-post mixed-methods study across a large Australian tertiary healthcare organisation examined handover page adoption using data from the EMR, and perceived usability and satisfaction were measured using a survey (handover page updated in EMR on 23 September 2024). Descriptive and inferential statistical analyses were conducted for quantitative data, and content analysis was used for qualitative data. **Results:** Adoption of the handover page was not statistically significant post-update (Wilcoxon signed-rank test z = −1.376, *p* = 0.169). Improved usability of the updated handover page post-update was demonstrated by a statistically significant decrease in the need to navigate away from the page to find relevant clinical information during handover (Fisher’s Exact Test *p* = 0.042). Nurses’ satisfaction increased, indicated by statistically significant increases in two items of the End User Computing Satisfaction Scale (precise information (Mann–Whitney U = 963.50, *p* = 0.040); and sufficient information (Mann–Whitney U = 927.50, *p* = 0.034)). Free-text comments indicated adoption and acceptability of the updated handover page by nurses, although a gap remains in the practice process. **Conclusions:** A co-designed solution to update the handover page within the EMR had good usability and satisfaction among nurses. Updates or implementations to digital health technologies must be continuously evaluated by specialist informatics teams to ensure appropriate adoption, usability and satisfaction by nurses, and positive repercussions for patient safety.

## 1. Introduction

Clinical handover is a communication process involving the transfer of patients’ information and care accountabilities among clinicians [1]. The Introduction, Situation, Background, Assessment and Recommendation (ISBAR) handover framework is a systematic and standardised communication framework that aims to improve patient safety by reducing communication errors [1]. To support clear communication, patient safety, and quality of healthcare delivery, the ISBAR handover framework is recommended for use by the Australian Commission on Safety and Quality in Health Care [2], and has been adopted in various clinical settings across Australia and internationally (e.g., the United Kingdom and the United States of America) [1].

The introduction of the EMR system across a large public Victorian tertiary healthcare organisation in 2019 provided an opportunity to reinforce a structured digital approach to nurses’ clinical handover. Pre-implementation of the EMR, the ISBAR framework was used across the healthcare organisation; however, ward-specific differences in its utilisation were observed for both paper and verbal handovers, despite the potential for verbal and written handovers to lead to poor information retention and communication mistakes [3]. A standardised ISBAR format for clinical handover was incorporated into the organisation-wide inpatient EMR system implementation as a page within the EMR designed to facilitate standardised and effective clinical handover between nurses. Utilising the EMR for clinical handover supports up-to-date, contemporaneous, centralised clinical information, reducing loss of information from verbal or paper-based clinical handover [4]. Clinical handover is a vital safety component of patient clinical documentation within Electronic Medical Record (EMR) systems [4,5]. However, there is limited evidence evaluating the impact of clinical handover within EMR systems throughout Australia [6].

Five years post its initial implementation, anecdotal evidence indicated that the electronic handover page was being poorly used and adopted by nurses. Across the organisation, multiple wards across sites were still utilising paper-based handovers or duplicating clinical handover information. To address this practice gap, an updated EMR handover page was developed by specialist informatics staff, in collaboration with a wide range of nursing staff. This provided an opportunity to reinforce approved nursing handover workflows and to reiterate the healthcare organisation’s principles for clinical documentation in the EMR: supporting contemporaneous documentation, avoiding paper-based patient documentation, and minimising duplication of information (including clinical handover). Minimising duplication of information supports nursing care delivery and patient care by utilising information already documented as part of care provision.

The aim of this study was to evaluate adoption, perceived usability, and satisfaction with the updated handover page within the EMR by nurses.

## 2. Materials and Methods

A pre-post mixed-methods study (concurrent embedded design) was developed to address the study aim and provide a broader perspective and interpretation of the pre- and post-handover page update impact(s) on nurses [7]. Quantitative and qualitative data were combined to form the overall study interpretation (discussion and conclusions).

Handover page adoption before and after the update was measured using data extracted from the EMR on the completion rates of handover documentation. Completion rate was calculated by dividing the number of completed handover entries by the minimum expected number of handover events, estimated using ward-level admission data. Handover page perceived usability and satisfaction were measured using a pre-post survey. Both the handover documentation completion rates and survey data collection were conducted at two time points: time point one was two months prior to the handover page update that occurred on 23 September 2024 (22 July 2024–22 September 2024); and time point two was two months post-handover page update (24 September 2024–24 November 2024).

### 2.1. Handover Update Project Team Development and Engagement Activities

To ensure the updated page was fit for purpose across the organisation, a specialist informatics project team was established, consisting of the Nursing and Midwifery Informatics team, EMR Clinical Applications team members, and EMR Change and Transformation Manager. The Exploration, Preparation, Implementation and Sustainment (EPIS) framework guided the EMR handover page update [8]. The Nursing and Midwifery Informatics team was key to this work to ensure relevant and timely improvement activities. This included conducting formal and informal engagements with nursing executives, nursing education teams, nurse managers, and nurses working across the healthcare organisation’s eight hospitals. An investigation of the handover page was conducted to determine what components are relevant to nursing handover, standardise practice across sites, and improve page relevance to nurses. Incentivising nurses to document contemporaneously drives the benefit of an automatically updated handover page, saving nurses’ time as they do not need to duplicate information to populate a separate handover sheet. Pre-update engagement activities occurred over four months and included focus groups and presentations at ward-, site-, and organisation-wide levels. Questions about the adoption, perceived usability, and satisfaction with the previous handover page were asked during the Nursing and Midwifery Informatics teams’ routine activities across sites.

### 2.2. Ward Selection

Representative wards were selected using a stratified random sampling approach from 71 eligible wards across the healthcare organisation. The wards were classified as either medical/surgical (n = 33), emergency (n = 5), intensive care (n = 7), paediatrics (n = 6), subacute (n = 9), or mental health (n = 11). The categories were independently confirmed by two members of the research team. Computer-generated random numbers using a stratified random sampling approach on a password-protected Microsoft Excel workbook were used to randomly select five wards for the pre- and post-update survey (three medical/surgical, one subacute, and one critical care), and ten wards to assess adoption (five medical/surgical, two intensive care, two subacute, and one paediatric). Power calculations were not conducted. The number of wards for both components was chosen as representative and feasible for data collection, with consideration to nursing staff’s competing priorities (e.g., other research and clinical care).

Other clinical areas across the healthcare organisation use the handover page within EMR but have variable admissions and nursing shifts (and therefore handover times), and were therefore excluded (radiology, operating theatre, pre-admission ward, day surgery ward, endoscopy, catheterisation lab, sleep centre, haemodialysis, and chemotherapy day unit).

Other areas are still documenting on paper, or using a hybrid model due to EMR system limitations (mixed use of EMR documentation and paper documentation), and were also excluded (mental health, maternity, outpatient clinics, community services, and clinical trials).

### 2.3. EMR Data and Expected Handover Events

At time point one, completion of handover between nurses was documented within the interactive view section of the EMR. At time point two, handover completion documentation was completed either within the interactive view section of the EMR or through the handover page itself (which then automatically populates the interactive view section of EMR; however, the reporting is separate).

The total number of patients per ward for the two time points was used to calculate the minimum number of expected handover events (calculated using the number of beds per ward assumed full, multiplied by the number of handovers per bed each day). This minimum number of expected handover events was then used as the denominator to calculate the percentage of handover documentation completed at time point one and time point two using the EMR data.

### 2.4. Survey

Nurses working on the five randomly selected wards were eligible to complete the survey at both time points. Three questions assessed nurses’ perceived adoption of the handover page using a 5-point Likert scale, asking about their use of the page, if they need to navigate away from the page during handover to find clinical information in other parts of EMR, and use of any paper (Never, Rarely, Sometimes, Often, or Always). These questions were developed by the healthcare organisation’s Nursing and Midwifery Informatics leadership team. Nurses’ satisfaction was evaluated using the End User Computing Satisfaction Scale [9], which included 12 questions and used a 5-point Likert scale (Strongly disagree, Disagree, Neither agree nor disagree, Agree, or Strongly agree). The questions were adapted by changing the word ‘system’ in the questions to ‘handover page’. Brief, non-identifiable demographics information was also collected, including age, years of nursing experience, hours worked per week, clinical speciality and nursing position. SPSS version 30 was used for statistical analysis of quantitative data (including descriptive and inferential statistics).

To protect staff anonymity and confidentiality, support participation, and minimise bias, the non-identifiable survey was hosted on the healthcare organisation’s server (survey completed in Microsoft Forms). Nurses were approached in person during the Nursing and Midwifery Informatics team’s regular rounding activities, and emails were sent to the five wards’ Nurse Managers for forwarding to their staff (convenience sampling). This protected nursing staff anonymity (by not obtaining individual email addresses) and distribution of surveys has been utilised for previous research studies within the healthcare organisation. Nurses were eligible to participate in the survey if they performed clinical intra-disciplinary handover as part of their duties. This included Enrolled Nurses, Registered Nurses, Clinical Nurse Specialists, Clinical Support Nurses/Clinical Educators and Associate Nurse Managers. All other staff were excluded.

A free-text comment box was supplied at the end of the survey for nurses to provide any other comments related to the handover page within the EMR. Inductive content analysis was used for the qualitative data [10]. At both the pre- and post-update stages, free-text comments were developed into concepts through discussion by three research team members (RJ, JG, and JS). A fourth team member was available to resolve discrepancies (was not required). Upon completion of qualitative coding (both pre- and post-update), a discussion was held with the broader research team to discuss the qualitative analysis.

### 2.5. Ethics Approval

Ethics approval was granted by Monash Health’s Human Research Ethics Committee (Reference: RES-24-000611Q, granted 5 August 2024). Informed consent for participation was obtained from all the subjects involved in the study.

### 2.6. Reporting Checklist

The Standards for Reporting Implementation Studies (StaRI) checklist was used to guide reporting of this study [11] (presented in Supplementary Material File S1).

## 3. Results

### 3.1. Adoption of Updated Handover Page

EMR data on actual and expected handover event results were used to assess handover page adoption. A Wilcoxon signed-rank test indicated there was no significant difference between handover documentation completion rate pre- and post-handover page update, z = −1.376, *p* = 0.169.

Table 1 presents the percentage of handover documentation completion in EMR pre- and post-handover page update. Table S1 in Supplementary Material File S2 includes the documentation of handover completion in EMR pre- and post-handover page update.

### 3.2. Survey Results

A total of 108 nurses completed the survey: 61 at time point one and 47 at time point two. Five survey responses had greater than 10% incomplete response fields and were, therefore, removed from analysis (three from time point one and two from time point two) [12]. Three more survey responses were excluded from time point one (participants working in excluded areas), leaving a total of 100 responses analysed: 55 at time point one and 45 at time point two.

Testing of Normality (using the Kolmogorov–Smirnov test) was undertaken for all the survey variables (including demographics). All the variables returned a statistically significant *p*-value of <0.001 (indicating violation of the assumption of normality); therefore, non-parametric statistical tests were used.

#### 3.2.1. Demographics

At both time points the majority of respondents were aged 20–29 (pre n = 22 (40.00%), post n = 19 (42.22%)); worked part-time (32 h per week) (pre- n = 26 (47.27%), post- n = 13 (28.89%)); and were Registered Nurses (pre- n = 43 (78.18%), post- n = 34 (75.56%)). Mann–Whitney U tests were run to assess for differences between nurses’ age, years of nursing experience, and hours worked per week in the pre- and post-handover page update groups. There was no significant difference between the nurses’ age pre- and post-handover page update, U = 1175.50, *p* = 0.689; and no significant difference between hours worked per week pre- and post-handover page update, U = 1000.00, *p* = 0.163. The survey respondents had fewer years of nursing experience pre-handover page update (n = 17, 30.91%) than post-handover page update (n = 14, 31.11%); however, this difference was not statistically significant (U = 1431.00, *p* = 0.073). Table 2 presents the nurse demographics data and Mann–Whitney U test results.

A chi-square test was performed to assess any significance between nursing positions in the pre- and post-update groups; however, the assumption of expected cell counts was violated (10 cells, 71.4%, had expected count less than 5, the minimum expected count was 0.45). Therefore, Fisher’s Exact Test was performed (*p* = 0.089), indicating a non-statistically significant difference between groups.

#### 3.2.2. Nurses’ Perceived Usability

Chi-square tests were undertaken to assess for significance in survey data on handover page perceived usability questions between pre- and post-groups (presented in Table 3). There was a statistically significant decrease in the need to navigate away from the updated page to find relevant clinical information during handover (demonstrating improved usability) (Fisher’s Exact Test *p*-value 0.042).

#### 3.2.3. Nurses’ Satisfaction

Mann–Whitney U tests were used to assess nurses’ satisfaction with the updated handover page. An increase in mean rank was observed in all twelve questions, of which two were statistically significant (updated handover page providing precise information and sufficient information). The Mann–Whitney U test results for the End User Computing Satisfaction Scale are presented in Table 4

#### 3.2.4. Free-Text Comments

Ten nurses provided free-text comments at the end of the pre-handover page update survey (18%), and nine nurses provided free-text comments at the end of the post-handover page update survey (20%). The demographics of the nurses who provided free-text comments (detailed in Table 5) indicate a range of nurses’ ages, years of experience, and clinical specialities.

Discussion with the broader research team at both time points supported the established concepts and did not yield any changes or additions to the content analysis. Coding inductively developed into a hierarchical structure at both time points.

**Figure 1:**
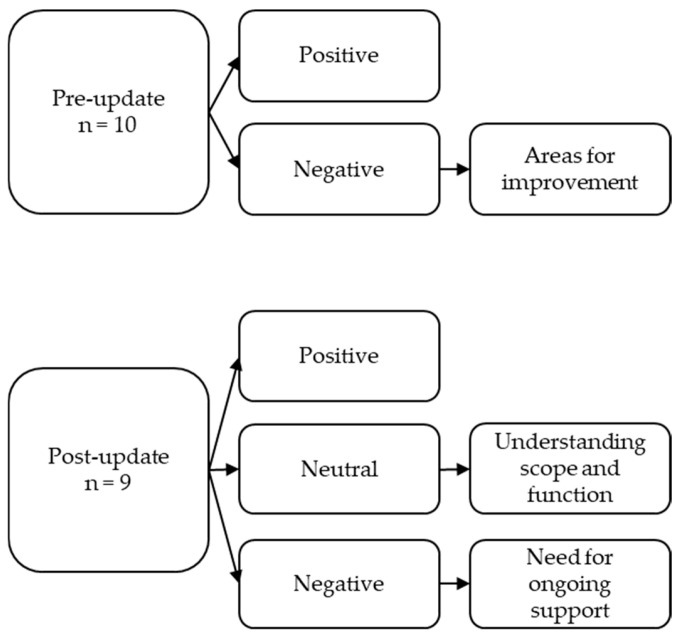
Qualitative coding framework.

Prior to the handover page update, comments were developed into two concepts: (i) satisfaction with the page, and (ii) areas for improvement (Figure 1).

(i). Satisfaction with the page

Several nurses were satisfied with the page content (“Clinically friendly tool” survey respondent (SR) 56; “It seems really good and easy to use” SR 33; and “very structured way of communication across different team members to enhance patient safety and reduce communication errors” SR 55). However, other nurses were looking forward to the handover page update (“need an [update]” SR 49 and “look forward to the system upgrade” SR 9).

(ii). Areas for improvement

Nurses had specific examples of what they would like optimised with the handover page update, including the overall layout (“Could use a better layout…easier to read” SR 50) and medications section (“Medication section…hard to visualise just only list of meds” SR 21). Two nurses also provided comments relating to frustration about the need to navigate away from the page during handover to access information in other sections of the EMR (“Also challenging when the other nurse is requesting to click out onto something else” S 9; “Some important info is not on the [page], I have to go through the documentation section to find out further” SR 3).

Post-page update, free-text comments were developed into three concepts: (i) positive feedback; (ii) understanding the scope and function of the handover page; and (iii) need for ongoing support for optimal page use (Figure 1).

(i). Positive feedback

Nurses’ positive experiences of the updated handover page related to minimisation of documentation duplication, which encouraged correct clinical documentation (as these components auto-populate the handover page) “It’s a great tool for handover” (post SR 16). Nurses also commented positively on the improved visibility of clinical components “I do like the view in the patient’s fluid status and line and devices” (post SR 37).

(ii). Understanding the scope and function of the page

Other comments related to requesting changes beyond both the scope of this research and the capabilities of the EMR system handover page, including asking for the research team to improve on incomplete or multi-disciplinary documentation (“please improve on…incomplete [documentation]” post SR 33; “Presenting complaints are most often not relevant or filled out accurately” post SR 10) and “better visual effects” (post SR 44). Another comment related to wanting to be able to edit clinical information from the handover page (“You cannot edit some information or add on” post SR 36); however, the page was designed to only pull data that has been completed elsewhere within the EMR (not changed with handover page update).

(iii). Need for ongoing support for optimal page use

Several comments included suggestions related to page use and usability that were already in place, e.g., describing scrolling within sections as “difficult” (post SR 33), and requesting hyperlinks that already exist on the updated page “have a link that take us directly to the pathology results” (post SR 37).

Comments relating to visibility and amount of information were contradictory, with one nurse wanting more patient information visible at one time (“there is not enough room for patients that have been there for some time”, post SR 36), whilst another described the page as a lot of information “making the person receiving the information overwhelmed” (post SR 44). Another nurse requested patient identifiers visible on the page “There are no 3 identifiers at the handover communication section to refer to” (post SR 33); however, these are already always visible at the top of the EMR. Limits on the default amount of information to be pulled into the page were decided upon during organisational consultation and required considerations of several factors, including page loading time, clinical relevance, and page flow.

## 4. Discussion

This study evaluated nurses’ adoption, perceived usability, and satisfaction with an updated handover page (using standardised ISBAR format) within the EMR. By reporting on the development and evaluation of a co-designed solution for the organisation-wide EMR with nursing staff, this work provides crucial evidence to help fill the gap in published literature related to nursing handover within the EMR [6].

Despite no statistical improvement in adoption (measured by an increase in handover documentation rates within the EMR), both quantitative and qualitative data from nurses reported improved perceived usability and satisfaction using the updated patient handover page within the EMR. Our study helped identify reported barriers to optimal handover page use by nurses, including knowledge on how to effectively use the page and its links. Statistically significant results in improved handover page perceived usability and satisfaction by nurses differ from a recently published rapid evidence assessment [6]. However, it is important to note that none of Browning et al.’s included studies were Australian, and the authors identified the lack of co-design, use of a validated tool, and pre- and post-assessment as gaps and limitations, all of which were addressed in this study [6].

Our study findings relating to nurse satisfaction further build on previous literature examining use of the standardised ISBAR format for nursing handover within the EMR [5]. Furthermore, our study helps fill an identified gap in research in the pre-post evaluation of a communication tool within the EMR [13].

Updates to the handover page were well received by nursing staff, indicated by positive qualitative comments. Specific responses discussed the benefit of having pre-existing patient identification fields within the EMR being visible whilst viewing the handover page. Qualitative feedback from nurses also indicated the need to continuously inform and reinforce design decisions made with the updated page to support care delivery across the healthcare organisation, which includes all patient ages. Feedback on the desire to customise or change views within the page highlights the need for further support or direction to available resources. This may help nurses understand how to best utilise page features, which may lead to increased perceived usability and satisfaction.

Overall, the findings of this study support the use of co-design and handover standardisation to address nursing handover within the EMR. Enabling the Nursing and Midwifery Informatics Team to lead this key workflow and engage accordingly enabled this co-design approach and ensured all the clinical areas were represented [14,15,16]. Ensuring stakeholder engagement ensured the standardisation of information relevant to nurses across the organisation, and supported development of an agreed minimum standard for handover information documentation to minimise barriers to use and adoption [17]. This study contributes to the nursing literature on how EMR systems must be continuously assessed post-implementation, and ongoing assessment must include end users in its co-design. This helps ensure nurses’ cognitive loads and possible factors that may negatively impact adoption, and communication in this study, are minimised as much as possible [6,18,19]. The use of an EMR for nursing handover is an international occurrence, and the potential impacts for nurses and patient safety related to handover adoption, perceived usability and satisfaction are, therefore, not limited to this organisation [1,4,5,6,18,19,20,21,22].

### Limitations

This study included several limitations, including the survey response rate, ward selection implications, and data on patient participation in, and perspectives of, handover. The study setting (and therefore impact on the generalisability of the results) must also be acknowledged. In-patient settings across the healthcare organisation include patients of all ages, including neonates, paediatrics, and adults. The survey response rate and subsequent limited qualitative data from free-text comments are important limitations to note. Randomisation of wards was undertaken to minimise bias; however, this means generalisability of our results must be carefully considered. To protect patient and staff anonymity, bed numbers were assumed to be full for each day throughout the study periods to calculate the expected number of handover events. Despite it being an important component of patient care delivery, patient participation in handover was not included in this study due to a concurrent collection of this data by another team. The research team decided it would unnecessarily burden patients to answer questions related to their handover participation twice. Patient perceptions of nursing handover using the EMR were outside of this study’s aim, but it is an important topic for future research. Another limitation is the timing of data collection (two months’ pre- and post-update). This timing was deemed to be a representative and feasible plan to assess nurses’ use, adoption, and satisfaction with handover within EMR; however, ongoing usability and satisfaction with the EMR handover page must, therefore, be assessed. A plan for this assessment is in place by the research team.

## 5. Conclusions

This study provides desperately needed research that supports evidence-based handover quality and efficiency with the development and evaluation of a co-designed solution with nursing staff for the organisation-wide EMR. There is a need for a specialist informatics team to continuously evaluate digital health technology interventions to ensure their impact on nurses and their work does not have any unintended consequences. All changes to digital health technologies, especially EMRs, must contribute to patient safety.

## Figures and Tables

**Table 1:** Percentage of handover documentation completion in EMR, per ward, pre- and post-handover page update.

	Ward A n (%)		Ward B n (%)		Ward C n (%)		Ward D n (%)		Ward E n (%)		Ward F n (%)		Ward G n (%)		Ward H n (%)		Ward I n (%)		Ward J n (%)	
	Pre	Post	Pre	Post	Pre	Post	Pre	Post	Pre	Post	Pre	Post	Pre	Post	Pre	Post	Pre	Post	Pre	Post
Number of beds	32		12		30		27		48		28		20		26		32		32	
Minimum number of handover events ^	5952	5856	1488	1464	5580	5490	3348	3294	8928	8784	5208	5124	3720	3660	4836	4758	5952	5856	5952	5856
Actual documented handover events	4518	4636	1422	1493	5367	5202	3570	3327	2663	2952	4769	4687	3408	3068	3999	3901	5992	5855	3619	3481
% handover documentation completed ^#^	75.91	79.17	95.56	101.98	96.18	94.75	106.63	101.00	29.83	33.61	91.57	91.47	91.61	83.83	82.69	81.99	100.67	99.98	60.80	59.44

^ Minimum number of handover events calculated by multiplying bed numbers by handovers per day by number of days in the pre- or post-handover update period. ^#^ Possible to have >100% handover documentation completed rate due to multiple instances of documentation of clinical handover within a shift.

**Table 2:** Nurse demographics data (survey respondents).

		Pre N = 55		Post N = 45		Mann–Whitney U test		
		n	%	n	%	U	Z	*p*-Value (2-Tailed)
Age	20–29	22	40.00	19	42.22	1175.50	0.400	0.689
	30–39	14	25.45	11	24.44			
	40–49	12	21.82	8	17.78			
	50+	3	5.45	6	13.33			
	Missing	4	7.27	1	2.22			
Years of nursing experience	0–1	17	30.91	6	13.33	1431.00	1.792	0.073
	2 to 5	14	25.45	14	31.11			
	6 to 10	11	20.00	9	20.00			
	11 to 15	5	9.09	5	11.11			
	16 to 20	3	5.45	4	8.89			
	21 to 25	5	9.09	4	8.89			
	>26	0	0.00	1	2.22			
	Missing	0	0.00	2	4.44			
Hours worked per week	8	0	0.00	2	4.44	1000.00	−1.394	0.163
	16	0	0.00	2	4.44			
	10	1	1.82	2	4.44			
	24	7	12.73	5	11.11			
	28	1	1.82	3	6.67			
	30	1	1.82	2	4.44			
	32	26	47.27	13	28.89			
	36	5	9.09	9	20.00			
	40	12	21.82	6	13.33			
	48	1	1.82	0	0.00			
	Missing	1	1.82	1	2.22			
Clinical speciality (multi-select)	Medical	19	N/A	15	N/A	N/A		
	Surgical	19		10				
	Speciality	13		23				
	Subacute	13		3				
	Mental health	1		0				
	Community	1		0				
	Intensive Care	1		0				
	Other	3		4				
Nursing position	Registered Nurse	43	78.18	34	75.56	N/A		
	Clinical Nurse Specialist	1	1.82	6	13.33			
	Associate Nurse Manager	8	14.55	4	8.89			
	Clinical Support Nurse or Educator	1	1.82	0	0.00			
	Graduate Registered Nurse	1	1.82	0	0.00			
	Enrolled Nurse	1	1.82	0	0.00			
	Nurse Manager	0	0.00	1	2.22			

N/A = Not applicable.

**Table 3:** Chi-square results for perceived usability of updated handover page questions.

	Pearson Chi-Square	df	*p*-Value (2-Sided)	Fisher’s Exact Test	
				Statistic	*p*-Value (2-Sided)
Use of page for clinical handover	9.265	4	0.055	N/A	
Navigate away during handover	Assumption of expected cell counts was violated (4 cells, 40.00%, had expected count less than 5, minimum expected count was 1.80)			9.323	0.042 *
Use of paper (other than approved list)	5.190	4	0.268	N/A	

* *p*-value < 0.05. N/A = Not applicable.

**Table 4:** End User Computing Satisfaction Scale results.

End User Computing Satisfaction Scale Question	Concept	Mean Rank		Mann–Whitney U	Z	*p*-Value (2-Tailed)
		Pre (N = 55)	Post (N = 45)			
1. Handover page provides precise information	Content	45.52	56.59	963.50	−2.051	0.040 *
2. Handover page provides content that meets my needs		46.00	56.00	990.00	−1.801	0.072
3. Handover page provides content that is beneficial for users in obtaining information		48.71	52.69	1139.00	−0.733	0.464
4. Handover page provides sufficient information		44.68	56.39	927.50	−2.114	0.034 *
5. The information contained in the handover page is accurate	Accuracy	46.65	55.21	1025.50	−1.605	0.109
6. I am satisfied with the accuracy of the handover page		47.65	53.99	1080.50	−1.171	0.242
7. Handover page is presented in a useful format	Format	47.82	53.78	1090.00	−1.076	0.282
8. Handover page presents clear information		47.16	54.58	1054.00	−1.355	0.175
9. Handover page is user-friendly	Ease of use	49.09	52.22	1160.00	−0.574	0.566
10. Handover page is easy to use		45.95	55.06	987.50	−1.674	0.094
11. Handover page provides what I need in time	Timeliness	46.43	55.48	1013.50	−1.616	0.106
12. Handover page provides up-to-date information		47.15	54.60	1053.00	−1.337	0.181

* *p*-value < 0.05.

**Table 5:** Nurse demographics data (free-text comments respondents).

		N = 19
		n
Age	20–29	6
	30–39	5
	40–49	4
	50+	3
	Missing	1
Years of nursing experience	0–1	4
	2 to 5	5
	6 to 10	4
	11 to 15	2
	16 to 20	2
	21 to 25	1
	>26	1
Hours worked per week	24	2
	32	10
	36	4
	40	3
Clinical speciality (multi-select)	Medical	6
	Surgical	6
	Speciality	10
	Subacute	3
	Other	1
Nursing position	Registered Nurse	14
	Clinical Nurse Specialist	1
	Associate Nurse Manager	2
	Graduate Registered Nurse	1
	Nurse Manager	1

## Data Availability

The raw study data are available upon reasonable request from the corresponding author (ethical reasons).

## Supplementary Materials

The following supporting information can be downloaded at https://www.mdpi.com/article/10.3390/nursrep15100369/s1, File S1: Standards for Reporting Implementation Studies: the StaRI checklist for completion [11,23]. File S2: Table S1. Documentation of handover completion in EMR, per ward, pre- and post-handover page update.

## Public Involvement Statement

No public involvement in any aspect of this research.

## Guidelines and Standards Statement

This manuscript was drafted against the Standards for Reporting Implementation Studies (StaRI) checklist.
