# Supplementary material for: Nurses’ Adoption, Perceived Usability, and Satisfaction with an Updated Electronic Handover Page Within the Electronic Medical Record: A Mixed-Methods Study
Source: Nurs Rep. 2025 Oct 15;15(10):369. doi: 10.3390/nursrep15100369 (PMC12567379; doi:10.3390/nursrep15100369)
Supplement: Supplementary file 1 [file nursrep-15-00369-s001.zip › Supplementary Materials File S2.pdf]

Supplementary Material File S2—**Table S1.** Documentation of handover completion in EMR, per ward, pre- and post-handover page update.

	Ward A n (%)		Ward B n (%)		Ward C n (%)		Ward D n (%)		WARD E n (%)	
	PRE	POST	PRE	POST	PRE	POST	PRE	POST	PRE	POST
Handover documentation = Yes	4518 (78.05)	4636 (79.82)	1422 (67.27)	1493 (67.16)	5367 (77.27)	5202 (77.77)	3570 (70.00)	3327 (69.89)	2663 (79.00)	2952 (78.28)
Handover documentation = No	3 (0.05)	5 (0.09)	4 (0.19)	2 (0.09)	2 (0.03)	3 (0.04)	3 (0.06)	2 (0.04)	1 (0.03)	3 (0.08)
Handover documentation = Blank	1268 (21.90)	1167 (20.09)	688 (32.54)	728 (32.75)	1577 (22.70)	1484 (22.19)	1527 (29.94)	1431 (30.06)	707 (20.97)	816 (21.64)
Total Handover documentation (includes Yes, No and Blank)	5789	5808	2114	2223	6946	6689	5100	4760	3371	3771

	WARD F n (%)		WARD G n (%)		WARD H n (%)		WARD I n (%)		WARD J n (%)	
	PRE	POST	PRE	POST	PRE	POST	PRE	POST	PRE	POST
Handover documentation = Yes	4769 (84.78)	4687 (83.37)	3408 (86.19)	3068 (82.87)	3999 (77.23)	3901 (75.81)	5992 (90.77)	5855 (91.47)	3619 (91.95)	3481 (93.05)
Handover documentation = No	2 (0.04)	2 (0.04)	0 (0)	0 (0)	1 (0.02)	0 (0)	2 (0.03)	2 (0.03)	2 (0.05)	2 (0.05)
Handover documentation = Blank	854 (15.18)	933 (16.60)	546 (13.81)	634 (17.13)	1178 (22.75)	1245 (24.19)	607 (9.20)	544 (8.50)	315 (8.00)	258 (6.90)
Total Handover documentation (includes Yes, No and Blank)	5625	5622	3954	3702	5178	5146	6601	6401	3936	3741
